# Supplementary material for: Bilateral Breast Gradient Insert Prototype for Strong Diffusion Encoding at 3 T
Source: Magn Reson Med. 2026 Oct 17;97(1):414–24. doi: 10.1002/mrm.70612 (PMC13746061; doi:10.1002/mrm.70612)
Supplement: Supplementary file 1 [file MRM-97-414-s001.docx]

Supplementary information

Figure S1: Breast volume distribution of the Duke breast MRI dataset. The total segmented breast volume (sum of both breasts) was divided by two to estimate the mean single-breast volume. The 91st percentile, which was used as the design cutoff for the breast gradient, is indicated.

Figure S2: Wiring pattern of the breast gradient at different angles.

Figure S3: Heating and cooling performance fitting of sensor 4 (integrated in the epoxy of the breast gradient close to Litz wire hotspot). Fits are indicated by red and blue. Fit parameters are shown in the table on the right.

Figure S4: Heating and cooling performance fitting of sensor 0 (located at the bottom of the breast cavity). Fits are indicated by red and blue. Fit parameters are shown in the table on the right.

Figure S5: Inductance and resistance of the breast gradient insert versus frequency.
